# Molecular Mechanisms of Gastrointestinal Stromal Tumors and Their Impact on Systemic Therapy Decision

**DOI:** 10.3390/cancers15051498

**Published:** 2023-02-27

**Authors:** Mojca Unk, Barbara Jezeršek Novaković, Srdjan Novaković

**Affiliations:** 1 Faculty of Medicine, University of Ljubljana, Vrazov trg 2, 1000 Ljubljana, Slovenia; 2 Division of Medical Oncology, Institute of Oncology Ljubljana, Zaloška 2, 1000 Ljubljana, Slovenia; 3 Department of Molecular Diagnostics, Institute of Oncology Ljubljana, 1000 Ljubljana, Slovenia

**Keywords:** GIST, KIT, PDGFRA, mutations, targeted therapy

## Abstract

**Simple Summary:**

Gastrointestinal stromal tumors (GISTs) are rare malignancies of the gastrointestinal tract recognized by their clinical presentation and using specific immunohistochemical staining for CD117 and DOG1. In recent years, prognoses of GISTs patients have significantly improved due to the introduction of tyrosine kinase inhibitors (TKIs) in clinical practice. *KIT/PDGFRA*-activating mutations are major driver alterations in the development of GISTs, leading to ligand-independent activation of KIT/PDGFRA receptors. The activated KIT/PDGFRA receptor is sensitive to the TKI imatinib. However, not all GIST patients respond to imatinib. Precise characterization of the driver mutations in GISTs—in particular, in the *KIT* and *PDGFRA* genes—and an understanding of the molecular mechanisms underlying resistance to imatinib and other TKIs should allow clinicians to select the most effective targeted drug as part of regular clinical practice. The correct choice of the TKI in the sequence of targeted agents should lead to improved survival for metastatic patients.

**Abstract:**

Gastrointestinal stromal tumors (GISTs) are soft tissue sarcomas that mostly derive from Cajal cell precursors. They are by far the most common soft tissue sarcomas. Clinically, they present as gastrointestinal malignancies, most often with bleeding, pain, or intestinal obstruction. They are identified using characteristic immunohistochemical staining for CD117 and DOG1. Improved understanding of the molecular biology of these tumors and identification of oncogenic drivers have altered the systemic treatment of primarily disseminated disease, which is becoming increasingly complex. Gain-of-function mutations in *KIT* or *PDGFRA* genes represent the driving mutations in more than 90% of all GISTs. These patients exhibit good responses to targeted therapy with tyrosine kinase inhibitors (TKIs). Gastrointestinal stromal tumors lacking the *KIT/PDGFRA* mutations, however, represent distinct clinico-pathological entities with diverse molecular mechanisms of oncogenesis. In these patients, therapy with TKIs is hardly ever as effective as for *KIT/PDGFRA*-mutated GISTs. This review provides an outline of current diagnostics aimed at identifying clinically relevant driver alterations and a comprehensive summary of current treatments with targeted therapies for patients with GISTs in both adjuvant and metastatic settings. The role of molecular testing and the selection of the optimal targeted therapy according to the identified oncogenic driver are reviewed and some future directions are proposed.

## 1. Introduction

Gastrointestinal stromal tumors (GISTs) are the most common mesenchymal neoplasms of the gastrointestinal tract, but they account for less than 1% of all gastrointestinal tumors. Incidence varies worldwide from 4.3/1 × 10^6^ inhabitants per year to 21.1/1 × 10^6^ inhabitants per year [1,2,3,4,5,6,7]. The incidence of GISTs is expected to be underestimated [8]. Most GISTs are indolent in their course and are discovered incidentally, but some are aggressive and disseminate early [8,9]. Gastrointestinal stromal tumors generally arise from pacemaker (Cajal) cells anywhere in the digestive tube—from the esophagus to the rectum—but, more recently, evidence has shown that they can also arise from telocytes or smooth muscle cells [10,11,12]. They are a heterogeneous group of diseases of different molecular subtypes, with oncogenesis mainly resulting from mutually exclusive activating mutations, most commonly in the *KIT* proto-oncogene (*KIT*) or the platelet-derived growth factor receptor alpha gene (*PDGFRA*) [13,14]. Around 5% of GISTs are syndromic and associated with germline mutations in genes encoding for KIT, PDGFRA, succinate dehydrogenase B/C/D (SDHB/C/D) (Carney Stratakis syndrome), and neurofibromin or with epigenetic silencing of *SDHC* (nonhereditary Carney triad syndrome) [15,16,17,18,19,20,21]. Patients confirmed to have GISTs with neurofibromin 1 (*NF1*) mutation or a deficient SDH complex should be referred to the outpatient clinic for genetic counseling.

Surgery is the only curative treatment for GISTs [22,23]. Improved understanding of the molecular biology of the disease and the identification of driver alterations and mechanisms of resistance to systemic therapies have resulted in advances in the systemic treatment of GISTs, thereby broadening the systemic therapy armamentarium. Tyrosine kinase inhibitors (TKIs) represent the standard systemic therapy and comprise various targeted drugs [22,23]. Chemotherapy is ineffective in patients with GISTs, and the time to disease progression with chemotherapy is less than 3 months [24]. Targeted therapy with the TKI of KIT/PDGFRA imatinib mesylate (imatinib) prolonged overall survival (OS) after surgery for high-risk GISTs [25]. Metastatic disease remains incurable; however, treatment with TKIs prolongs survival from 1.5 to over 5 years [22,23,26]. Despite the convincing achievements with TKI treatment, targeted therapy eventually leads to the development of drug resistance. Secondary mutations play a major role in this process, allowing for the selection of cells that are resistant to the treatment applied [27,28,29,30].

This review provides an outline of current knowledge on the molecular mechanisms of GISTs and the selection of the optimal systemic treatment according to the identified molecular mechanisms, both in limited and metastatic disease, and proposes future directions for research.

## 2. Molecular Classification of GISTs

**Figure 1:**
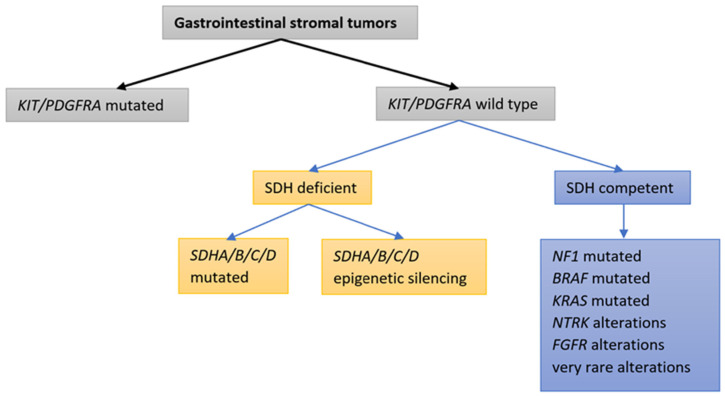
Classifications of GISTs based on tumor genetic alterations: molecular classifications.

The proto-oncogene *KIT* encodes the KIT receptor, which is a type III receptor tyrosine kinase (RTK); it belongs to a family of RTKs that also includes PDGFRA, platelet-derived growth factor receptor beta (PDGFRB), colony-stimulating factor 1 receptor (CSF1R), and Fms-like RTK 3 (FLT3). The receptor tyrosine kinase KIT (CD 117) is normally expressed in Cajal cells in the gastrointestinal tract. It plays an important role in the development of a normal pacemaker system in the gut [10]. In 1998, Hirota and colleagues discovered that activating *KIT* mutations are the major mechanism of GIST oncogenesis [14]. Activating mutations in *KIT* lead to the formation of a permanently active protein that is a target for imatinib binding. Of equal importance—and the second most common driver mutations in GISTs—are mutations in *PDGFRA*, which encodes the PDGFRA receptor tyrosine kinase. The PDGFRA RTK itself is homologous to the KIT RTK and, again, an activating mutation in *PDGFRA* results in the formation of a permanently active RTK that is also a target for imatinib. KIT/PDGFRA-induced oncogenesis mediates the rapidly accelerated fibrosarcoma (RAF)–mitogen-activated protein kinase (MEK)–mitogen-activated protein kinases (MAPK) (RAF-MEK-MAPK) and phosphatidylinositol 3-kinase (PI3K)/protein kinase B (AKT) (PI3K-AKT) signaling pathways [10,31,32]. At present, it is believed that *KIT/PDGFRA*-activating mutations are mutually exclusive [28]. In recent years, it has been discovered that only 85–90% of GISTs have an activating mutation in *KIT/PDGFRA*, while in the remaining 10–15%, the molecular mechanism of oncogenesis has not been determined (historical wild-type (WT) GISTs). With advances in molecular diagnostic techniques—in particular, with the use of next-generation sequencing (NGS) technology—the mechanisms of oncogenesis can be more precisely defined, even in historical WT GISTs. With the increased sensitivity of the NGS method, it has been discovered that driver mutations in *KIT/PDGFRA* are also the most frequent in historical WT GISTs. Taking this fact into account, *KIT/PDGFRA* mutations represent the driving mutations in as many as 92–93% of all GISTs [22,33,34,35,36,37,38]. In 5–7.5% of all GISTs, the driving oncogene mechanism is related to a deficiency in the succinate dehydrogenase (SDH) complex [16,18,19,28,39,40,41,42,43,44,45,46]. When no *KIT/PDGFRA* mutations are detected and the SDH complex is competent, various very rare driver alterations have been identified: alterations in the Rat sarcoma virus (*RAS*) gene family, the v-Raf murine sarcoma viral oncogene homolog B1 (*BRAF*) gene, the *NF1* gene, the neurotropic tyrosine receptor kinase 1–3 (*NTRK1–3*) genes, and the fibroblast growth factor receptor 1–4 (*FGFR1–4*) genes [18,28,33,39,40,44,47,48,49,50,51]. “True” WT GISTs for which advanced molecular techniques fail to demonstrate any driving alteration are very rare [33,34,35,36,37]. Figure 1 illustrates the principal molecular classifications of GISTs.

Different molecular mechanisms in GISTs lead to different clinical courses of the disease and, in particular, different responses to systemic imatinib treatment. Therefore, it is necessary to identify the driving alterations before initiating the first systemic therapy, both in (neo)adjuvant settings and as the first systemic therapy for metastatic disease [22,23].

## 3. Identifying the Molecular Mechanisms of GIST

Molecular analysis is an important factor influencing decision about systemic treatment for both limited and metastatic disease. Molecular markers have both prognostic and predictive value [52,53,54,55]. The two methods so far predominantly used to identify molecular markers are reverse transcription polymerase chain reaction (RT-PCR) and direct Sanger sequencing. Both RT-PCR and Sanger sequencing have their limitations, primarily reflected in the restricted testing for known driver changes (hot spot mutations) and in the time-consuming process of identifying driver changes in the selected gene. Even then, these methods are only successful in cases with a relatively high allele frequency for the altered gene. For Sanger sequencing, the detection limit is set at 20% of the altered deoxyribonucleic acid (DNA) in the sample. For these reasons, NGS, which has a substantially lower detection limit and allows for the identification of changes in a large number of genes in several samples simultaneously, is progressively entering routine diagnostics. Several studies have been published demonstrating that NGS outperforms RT-PCR and direct Sanger sequencing in its ability to identify molecular alterations in GISTs [34,48,49,50,51]. Next-generation sequencing also exhibits a higher positive predictive value than RT-PCR and direct Sanger sequencing [33,34]. The European Society of Medical Oncology (ESMO) guidelines for the management of patients with GISTs recommend centralization of molecular testing, although they allow either Sanger sequencing or NGS as the applied method. If no driver mutation in *KIT/PDGFRA/BRAF* is demonstrated, they advise immunohistochemical (IHC) staining to determine SDH B protein (SDHB) expression. In GISTs where no driver alteration in *KIT/PDGFRA/BRAF* is established and SDHB is expressed, they further recommend excluding driver alterations in *NF1* [22]. The National Comprehensive Cancer Network (NCCN) recommendations, on the other hand, are more specific: patients in whom sequencing fails to demonstrate mutation in *KIT/PDGFRA* and who have a competent SDH complex, as confirmed by IHC staining, should be tested with NGS to identify any targetable driver alterations in other genes (*BRAF*, *NTRK*, *FGFR*) [23].

### 3.1. GISTs with KIT or PDGFRA Mutations

The human KIT proto-oncogene is located on chromosome 4 (q12). This gene was discovered in 1987 [56]. A nonmutated KIT encodes a type III RTK. KIT protein is a transmembrane RTK with extracellular (EC), transmembrane (TM), and intracellular (IC) domains. The EC domain is composed of five Ig-like regions; three are responsible for stem cell factor (SCF) binding and the other two for protein dimerization after SCF binding. The TM helix domain links the EC to the IC domain; the IC domain is composed of a juxtamembrane domain (JMD) and a tyrosine kinase domain (TKD). The TKD is composed of a phosphotransferase domain (PTD), an adenosine triphosphate (ATP)-binding site, and an activation loop. The JMD part controls and regulates the function of TKDs [57]. Binding of SCF causes homodimerization of the two KIT RTKs, leading to autophosphorylation of the homodimer. This releases adenosine diphosphate (ADP) and binds ATP to the active site. Further phosphorylations of parts of the IC domain of the KIT RTK follow, and only when these are complete is KIT fully active [58]. Activating mutations in KIT are the major contributor to oncogenesis in GISTs since they lead to permanent activation of KIT protein without the need for prior SCF (ligand) binding. The majority (approximately 60%) of mutations in KIT are in exon 11, including deletions (the largest number between codons 550 and 560), deletions and insertions (indels), and insertions and missense mutations, resulting in structural changes in the JMD [32,38,58]. This region has an autoinhibitory function in kinase activation, which is reduced by the mutation. Less frequently (9–10%), mutations may occur in exon 9, which encodes the EC domain of KIT (mostly tandem duplications) [32,38,58]. Mutations in KIT exon 8 (EC domain) and in exons 13 and 14 (ATP binding site on TKD), and 17 (IC domain of activation loop) are very rare events in GIST oncogenesis [32,38].

Activated KIT triggers different signaling pathways: RAS/MAP/MAPK, PI3K/AKT, phospholipase C gamma (PLC-gamma), Janus kinase (JAK)/signal transducer and activator of transcription (STAT) (JAK/STAT), and Scr kinase pathways. Which pathway will be activated depends on which tyrosine residue of the IC domain is phosphorylated [57].

In humans, the proto-oncogene PDGFRA is also located on chromosome 4 (q12), in the same region as KIT, and encodes a class III RTK that is structurally homologous to KIT RTK. The activation of downstream pathways is also similar to KIT; PDGFRA activation predominantly activates the RAS/RAF/MAPK and PI3K/AKT signaling pathways [10,31,32]. In GIST, mutations of PDGFRA are more infrequent than mutations of KIT [32]. The greater number (up to 15% of all GISTs) of PDGFRA mutations affect exon 18 (the activation loop of the IC domain); less frequently (up to 2% of all GISTs), they may affect exon 12 (in the JMD) and, even less frequently (1%), exon 14 (the ATP binding site TKD) [38,57]. PDGFRA exon 18 D842V mutation is the most common PDGFRA mutation (50–70% of PDGFRA mutant GISTs and about 8% of all GISTs) and results in a stable conformational structure for tyrosine kinase (TK) in its active form [59].

#### 3.1.1. Targeted Therapy for GISTs with KIT or PDGFRA Mutations

Three years of adjuvant treatment with 400 mg/day imatinib in *KIT/PDGFRA*-mutated GISTs with high risk for disease recurrence prolongs recurrence-free survival (RFS) (ten-year RFS: 52.5% vs. 41.8%) and OS (ten-year OS: 79.0% vs. 65.3%) compared to 1 year of treatment alone [25,60,61]. A high estimated risk of recurrence (higher than 50%) is assessed according to the modified National Institutes of Health (NIH) consensus criteria [62,63]. The efficacy of adjuvant imatinib treatment depends on the type of *KIT/PDGFRA* mutation. Patients with *PDGFRA* mutations, *KIT* exon 11 duplications, insertions, and substitutions have longer RFS than patients with *KIT* exon 11 deletions or indel mutations. Patients with *KIT* exon 9 mutations (most commonly an AY duplication) have the shortest RFS. Patients with *KIT* exon 11 deletions or indels (especially if they affect codons 557 and/or 558) have a significantly longer RFS if treated with 3 years of imatinib compared to 1 year. This difference in RFS with regard to the duration of adjuvant imatinib treatment has not been observed in other groups (*KIT* exon 11 substitutions and *KIT* exon 9 mutations) [61]. Given the longer progression-free survival (PFS [64]) and better response to treatment in *KIT* exon 9-mutated metastatic patients with the 800 mg dose compared to the 400 mg/day dose, the question of an escalated dose of imatinib for *KIT* exon 9 mutation also arises in the context of adjuvant therapy. Results from a multi-institutional European retrospective study of patients with *KIT* exon 9-mutated GISTs treated with adjuvant imatinib revealed that a higher daily dose of 800 mg versus 400 mg did not improve survival outcomes (RFS: hazard ratio (HR), 1.24; mRFS: HR, 1.69; imatinib failure-free survival (IFFS): HR, 1.35; 95% CI, 0.79–2.28) [65]. However, no adjuvant prospective randomized studies with an escalated dose of imatinib in this population have been published so far [53,66,67].

Taking into consideration that a longer duration of adjuvant imatinib therapy leads to longer survival, another dilemma appears regarding further prolongation of adjuvant imatinib therapy beyond 3 years. In a phase 2 clinical trial involving imatinib-sensitive GISTs with 5 years of adjuvant imatinib therapy following resection in patients with a high risk of recurrence, the five-year RFS estimate was 90% and the five-year OS estimate was 95% [68]. Two more phase 3 clinical trials are currently underway comparing 3 versus 5 years of adjuvant imatinib and 3 versus 6 years of adjuvant imatinib for GISTs with a high risk of recurrence (NCT02413736 and NCT02260505). Currently, the standard adjuvant treatment is recommended for 3 years [22,23]. Table 1 shows the results of pivotal clinical trials employing imatinib as adjuvant therapy.

The type of *KIT/PDGFRA* mutation is not the only factor influencing adjuvant treatment efficacy. Other factors, such as the duration of adjuvant treatment, primary site (non-gastric primary sites being connected to poorer disease-free survival (DFS)), tumor size (larger tumor size being associated with a poorer DFS), mitotic index (high mitotic index being associated with poorer DFS), and female sex (an independent prognostic factor for a higher PFS and OS), have also been shown to contribute [52,69].

**Table 1:** Pivotal clinical studies with imatinib in the adjuvant setting.

Study First Author/Publication Information	Number of Patients, Patient Population	Clinical Phase	Intervention	Molecular Analysis	Primary Endpoint	Results
Dematteo et al., Lancet 2009 [70]	713 Patients resected for ≥3 cm GIST	3	1 y adjuvant imatinib 400 mg/d vs. placebo	No	RFS (primary endpoint changed from OS to RFS)	1 y RFS: 98% (imatinib) vs. 83% (placebo), HR 0.35. No OS benefits
Casali et al., JCO 2015 [71]	908 Patients after R0-1 surgery for localized high- or intermediate-risk GISTs according to NIH criteria [72]	3	2 y adjuvant imatinib 400 mg/d vs. no treatment	No	IFFS (primary endpoint changed from OS to IFFS)	5 y IFFS: 87.0% (imatinib) vs. 84.1% (control), *p* = 0.21, HR 0.79. No OS benefits
Joensuu et al., JAMA 2020 [25]	397 Patients resected for high-risk GISTs according to modified NIH criteria [62]	3	1 y vs. 3 y adjuvant imatinib 400 mg/d	Yes (366/397)	RFS	10 y RFS: 52.5% (3 y treatment) and 41.8% (1 y treatment), HR 0.66, *p* = 0.003 10 year OS: 79.0% (3 y treatment) and 65.3% (1 y treatment), *p* = 0.004, HR: 0.55.

R0—radical surgery, R1—surgery with positive microscopic margin, OS—overall survival, RFS—recurrence-free survival, NIH—National Institutes of Health, IFFS—imatinib failure-free survival, d—day, y—year, HR—hazard ratio.

The goal of systemic treatment of metastatic or unresectable *KIT/PDGFRA*-mutated GISTs with imatinib is to prolong survival with good quality of life. The initial dose of imatinib is 400 mg/day, except for patients where *KIT* exon 9 mutation is known to be the oncogenic driver; in these patients, treatment is started upfront with an escalated dose of 800 mg/day [67]. In most cases, the dose of 400 mg daily results in up to 5% complete response, 40–68% partial response, and 14–32% stable disease, with a PFS of approximately 40 months [24,66,73,74,75,76].

However, there is a group of patients who respond worse to imatinib initiation; namely, some patients with *KIT* exon 11 mutations who, at the same time, bear polymorphisms in genes involved in imatinib metabolism (reducing metabolic capacity), patients with *KIT* exon 9 mutations, patients without *KIT/PDGFRA* mutations (WT GISTs), and patients with primary resistance to imatinib, especially those carrying *PDGFRA* D842V and *KIT* D816V mutations [32,77,78]. Patients with *KIT* exon 9 mutation achieve a PFS of only 12.6–16.7 months with 400 mg/day imatinib [53,66,67]. However, an escalated dose of 800 mg/d of imatinib has been shown to be more effective in these patients, giving an objective response rate (ORR) of 47% (vs. 21%) and a better PFS (HR = 0.57; *p* = 0.017) but without improving the OS [53]. Patients with “true” WT GISTs do not respond to imatinib treatment because their tumors have no target to which imatinib would bind [34,66,67]. Primary resistance to imatinib is supposed to be a landmark of GISTs bearing *PDGFRA* D842V or *KIT* D816V mutations [79,80,81]. Nevertheless, cases of partial response to imatinib in patients with the *PDGFRA* D842V mutation have been described [34,37,82].

#### 3.1.2. Progression of GISTs with KIT or PDGFRA Mutations after the First-Line Targeted Therapy

Disease progression is frequently due to the occurrence of secondary mutations resulting from evolutionary selection pressure during imatinib treatment [83]. They occur in 85 to 90% of patients, with a median time to onset for secondary mutations—and, thus, disease progression—of 20–24 months [35,74,84,85]. Secondary mutations result in a modified RTK structure. As an outcome, imatinib is no longer effective, as no optimal binding sites remain for imatinib; thus, the inhibition of signaling via mutated RTK ceases. Continuous activation of RTK is re-established and the disease progresses.

In everyday clinical practice, we do not strive to confirm the exact mechanism of resistance due to the heterogeneity of mutations occurring at progression. If the progression is focal (a “nodule within a mass” up to one or a few nodule(s)/mass(es) while the rest of the disease is still responding), surgery or nonsurgical procedures (ablation, radiotherapy) may be selected [22,23]. When local ablative therapy is not feasible, second-line therapy with multitargeted TKIs is initiated.

Secondary mutations occur mainly at specific sites in *KIT* or *PDGFRA*. However, the response to second-line TKI treatment is not always the same and depends on the mechanism of resistance and the efficacy of the TKI applied. Secondary mutations, typically missense mutations, most commonly affect the coding region of the ATP binding site (exons 13 and 14 of *KIT* and exon 14 of *PDGFRA*), as well as the coding region of the TKD activation loop (exon 17 of *KIT* and exon 18 of *PDGFRA*) [30]. KIT/PDGFRA receptor tyrosine kinases with secondary mutations in regions encoding the ATP binding site are sensitive to treatment with sunitinib, ripretinib, avapritinib, and ponatinib but do not respond to treatment with regorafenib or sorafenib [86,87,88,89,90,91]. KIT/PDGFRA receptor tyrosine kinases with secondary mutations in genes encoding the activation loop are sensitive to regorafenib, ripretinib, avapritinib, sorafenib, nilotinib, and ponatinib [87,89,90,92,93,94,95].

Following disease progression with imatinib, the current guidelines recommend the use of three multitargeted TKIs—sunitinib, regorafenib, and ripretinib. There are also data in the literature on the efficacy of other multitargeted TKIs in this setting (clinical trials on the efficacy of sorafenib, dovitinib, masitinib, ponatinib, nilotinib, and pazopanib), but none of them exceeded a 6 month barrier for mPFS [22,23,96]. The prototype for multitargeted TKIs is sunitinib, a small molecule that inhibits the activity of multiple (over 80) RTKs involved in tumor growth, pathological angiogenesis, and malignant cell proliferation. Sunitinib inhibits PDGFRA and PDGFRB; vascular endothelial growth factor receptors 1, 2, and 3 (VEGFR1, VEGFR2, and VEGFR3); KIT; FLT3; CSF-1R; and glial neurotropic factor receptor (RET), among others [97]. Other multitargeted TKIs have similar but not identical spectra of activity.

The mechanisms of secondary mutations in *PDGFRA* are poorly understood, with well-known primary resistance to imatinib only in *PDGFRA* D482V (and its homologue *KIT* exon 17 mutation D816V) [32]. Avapritinib represents a new option for systemic therapy targeting *PDGFRA* D842V. In a phase 3 clinical study, it exhibited good efficacy and an objective response of 84% [90].

Other mechanisms of GIST resistance to imatinib are infrequent and poorly understood. Resistance could potentially be induced by activation of other oncogenic pathways—e.g., dysregulation of cyclin-dependent kinase inhibitor 2A (CDKN2A); loss of tumor protein 53 (TP53) function; inactivation of dystrophin; genomic alterations in chromosomes 1p, 14q, and 22q; and overexpression of KIT [47,59,98,99,100].

Table 2 shows pivotal clinical trials of targeted drugs recommended for systemic treatment of metastatic GISTs. Sunitinib exhibited moderate efficacy after progression with imatinib in a randomized phase 3 clinical trial (RCT) involving metastatic GISTs, with a significantly longer PFS (27.3 weeks vs. 6.4 weeks with placebo; HR 0.33; *p* < 0.0001) [86]. The efficacy of sunitinib is, however, not universal. It depends on the type of primary mutation and secondary mutation. In patients with the primary mutation in *KIT* exon 9, sunitinib elicits improved PFS in OS compared to those with the primary mutation in *KIT* exon 11 [101,102]. Furthermore, the type of secondary mutation influences PFS and OS [30,86,102]. In a phase 3 RCT, regorafenib prolonged median PFS after treatment with imatinib and sunitinib compared to placebo (4.8 months vs. 0.9 months; HR 0.27; *p* < 0.0001) [92]. The efficacy of regorafenib is again not universal as it depends on the type of secondary mutation [30,64]. In a phase 3 RCT, ripretinib prolonged median PFS after treatment with imatinib, sunitinib, and regorafenib compared to placebo (6.3 months vs. 1.0 month; HR 0.15; *p* < 0.0001). The efficacy of ripretinib is also not universal, and it depends on the type of secondary mutation [103]. In a phase 1 clinical trial, avapritinib employed for patients with unresectable GISTs and *PDGFRA* D842V mutations, regardless of prior systemic therapy, achieved an objective response in 88% of patients (9% complete response, 79% partial response) [90]. The efficacy of avapritinib in *KIT/PDGFRA*-mutated, imatinib-pretreated (*PDGFRA D842V* excluded) patients is only modest, with an ORR of 17%, median duration of response (mDOR) of 10.2 months, and mPFS of 3.7 months [104].

### 3.2. GISTs without KIT/PDGFRA Mutations

#### 3.2.1. GISTs without KIT/PDGFRA Mutations and with Deficient SDH Complex

The *SDHA* tumor suppressor gene (TSG) is located on chromosome 5 (p15.33), the *SDHB* TSG on chromosome 1 (p36.13), the *SDHC* TSG on chromosome 1 (q23.3), and the *SDHD* TSG on chromosome 11 (q23.1). They encode the four subunits (SDHA, SDHB, SDHC, and SDHD) of heterotetrametric enzyme SDH, the key enzyme in the Krebs cycle and respiratory chain in mitochondria.

The SDH enzyme complex catalyzes the oxidation of succinate to fumarate. Loss of function of mitochondrial SDH (due to mutations in the *SDHA*, *SDHB*, *SDHC*, or *SDHD* Miettinen et Lasotation of SDHB [41]. Succinate accumulates and inhibits the activity of dioxygenases (ten-eleven translocation methylcytosine dioxygenases (TETs) and histone lysine (K) demethylases (KDMs)). These enzymes degrade hypoxia-inducible factor 1a (HIF-1a) protein, which accumulates in the absence of dioxygenases and increases transcription of the genes it regulates: the insulin-like growth factor 1 receptor (*IGF1R*) and *VEGFR*. Furthermore, a lack of dioxygenase activity triggers hypermethylation of DNA; i.e., epigenetic silencing. Consequently, activation of IGF1R and VEGFR and/or an increase in DNA methylation lead to malignant transformation of normal interstitial Cajal cells into GISTs [21,39,107].

Deficiency in the SDH complex is a rare event in GISTs, present in up to 5–7.5% of GIST patients [16,18,19,28,39,40,41,42,43,44,45,46]. When the gene coding for any subunit is biallelically inactivated, IHC staining for SDHB is absent and shows only KIT and DOG1 [18,46]. Complex mutations in genes for subunits of the SDH complex are found in non-syndromic GISTs with a deficient SDH complex [46]. Since somatic changes are extremely rare, the absence of IHC staining for SDHB is highly likely to indicate syndromic disease caused by germline mutation [46]. Indeed, in half of patients with deficient SDH complexes, the trigger of the GIST is a germline-inactivating (“loss of function”) mutation of a gene encoding one of the SDH subunits (typically a germline *SDHA* mutation) in combination with a somatic mutation (“frameshift” deletion with stop codon, “missense”, “nonsense”, and “splice site” mutations) [16,46]. Germline mutations in genes coding for the SDHB/C or D subunits are associated with a rare hereditary Carney–Stratakis syndromic disease encompassing the dyad of a GIST and paraganglioma [16,17]. In the other half of the population of patients with deficient SDH complexes, the origin of the GIST is an epigenetic silencing of *SDHC* (post-zygotic hypermethylation of the promoter region). The specific hypermethylation pattern of the *SDHC* gene is associated with a rare non-hereditary Carney triad syndrome (a GIST, paraganglioma, and lung chondroma with a deficient SDH complex) [18,19,21,41,46,108].

##### Targeted Therapy for GISTs without KIT/PDGFRA Mutations and with a Deficient SDH Complex

Gastrointestinal stromal tumors with a deficient SDH complex do not respond to imatinib treatment but, in line with the mechanism of oncogenesis, they respond to multitargeted TKIs, which are potent antiangiogenetic agents, such as sunitinib, regorafenib, and pazopanib [23,40,43,97,109,110,111,112]. Linsitinib, the IGF1R inhibitor, is moderately effective in these patients [113]. Temozolomide (TMZ) has been shown to cause DNA damage and apoptosis in a pre-clinical study in patient-derived SDH-deficient GIST models [114]. Preliminary results from nine patients enrolled in a phase 2 clinical trial of TMZ demonstrated an ORR at 6 months of 22.2% and disease stabilization at 6 months in 22.2% [115]. A clinical phase 1 trial is presently enrolling patients with SDH-deficient GISTs to be treated with INBRX-109 (tetravalent death receptor 5 (DR5) agonist antibody) in combination with temozolomide (NCT03715933). A phase 2 clinical trial with rogaratinib is currently underway in metastatic GIST patients with a deficient SDH complex (NCT04595747).

#### 3.2.2. GISTs without KIT/PDGFRA Mutations and with a Mutation in Neurofibromin 1 (NF1)

The *NF1* gene is a huge TSG located on chromosome 17 (q11.2). It codes for the protein neurofibromin, which plays a role in the RAS/MEK/MAPK and mammalian target of rapamycin (mTOR) oncogenic pathways. Neurofibromin acts as a guanine triphosphate (GTP) hydrolase (GTPase), converting RAS-GTP to the inactive RAS guanosine diphosphate (GDP). Inactivating mutations in *NF1* result in the accumulation of RAS-GTP, followed by increased RAS signaling in the RAS/MEK/MAPK signaling pathway [116]. Germline *NF1* inactivation causes neurofibromatosis type 1 (NF1), a relatively common autosomal dominant genetic disorder characterized by predisposition to cancer development. Clinical studies have demonstrated that different inactivating mutations of *NF1* exhibit different phenotypic variants and clinical presentations [117]. Due to the highly variable phenotype of NF1, other genes (so-called modifier genes) are likely to be involved in the pathogenesis in addition to the *NF1* gene mutation [117]. Inactivating mutations of *NF1* in GIST patients have been described as “missense” mutations, “nonsense” mutations, “frameshift-induced” protein truncation, “splice site” mutations, and larger deletions [28,117,118,119,120,121]. It has been confirmed that up to 7% of patients with NF1 develop GISTs. However, patients with NF1 GISTs account only for approximately 1–2.4% of all patients with GISTs [38,44,116,118,120]. Neurofibromatosis type I-associated GISTs exhibit immunohistochemical expression of KIT, DOG1, and SDHB, frequently present with loss of heterozygosity at 14q and 22q and, occasionally, with *KIT* mutations and/or mutations in the Notch signaling pathway [121,122,123]. Loss of 14q and 22q heterozygosity correlates with early disease presentation [121]. As already stated above, NF 1 is a negative regulator of the RAS/MEK/MAPK signaling pathway and, thereby, inactivating mutations in the *NF1* gene result in increased signaling in this pathway independent of RTK KIT activation [119]. This explains the ineffectiveness of imatinib in patients with *NF1* mutations and the current lack of any effective systemic treatment for NF1 GISTs.

#### 3.2.3. GISTs without KIT/PDGFRA Mutations and with Mutations in BRAF

The human *BRAF* proto-oncogene is located on chromosome 7 (7q34). It encodes the BRAF protein, which is a serine threonine kinase with the function of activating the MAPK signaling pathway. Mutations in the *BRAF* gene are divided into three classes according to the impacts they have on the function of BRAF protein [124]. Class one involves mutations that allow BRAF to function as a constitutively active monomer. Class two involves mutations that allow the formation of a constitutively active dimer, and class three involves mutations that weaken or completely abolish the kinase activity of the BRAF protein. The greater part of the clinically relevant mutations in *BRAF* are mutations in exon 15, the most common of which is a substitution of valine with aspartate at codon 600 (*BRAF* V600E). This mutation leads to phosphorylation of the activation domain of the BRAF kinase and, consequently, its constitutive activation [125]. The *BRAF* V600E mutation is a driver mutation in various solid cancers, but it is a rare event in GIST tumorigenesis [126]. According to the literature, it occurs in less than 1% of adult GIST patients [125,127,128,129,130,131].

##### Targeted Therapy of GISTs without KIT/PDGFRA Mutations and with Mutations in BRAF

Gastrointestinal stromal tumors with *BRAF* V600E do not respond to treatment with imatinib and sunitinib, but a successful case of treatment with regorafenib has been described [132]. Treatment with dabrafenib (BRAF inhibitor), with or without trametinib (MEK inhibitor), has been proven successful as tumor agnostic therapy for solid cancers with a verified *BRAF* V600E mutation and is approved by the US Food and Drug Administration (FDA), but not by the EMA, as tumor agnostic therapy [133]. To date, one case of successful treatment of a metastatic *BRAF* V600E GIST with the BRAF inhibitor dabrafenib alone (without the MEK inhibitor trametinib) has been described [134]. There are no data on adjuvant treatment with BRAF/MEK inhibitors; therefore, adjuvant treatment is not recommended [22,23]. Additionally, other *BRAF* rearrangements (*PRKAR1B-BRAF*), the clinical significance of which is so far unknown, have been recently recognized [135,136].

#### 3.2.4. GISTs without KIT/PDGFRA Mutations and with Mutations in KRAS

The human *KRAS* proto-oncogene is located on chromosome 12 (12p11.1–12p12.1). It codes for KRAS protein, a hydrolase that converts the nucleotide GTP to GDP and is part of the RAS super-family of GTPases. Active KRAS has the GTP molecule bound and, under physiological conditions, allows transduction of signals downstream to the RAS/MEK/MAPK and PI3K/AKT signaling pathways. Mutations in *KRAS*, predominantly point mutations, can be either primary or secondary in GIST evolution. Both primary and secondary mutations result in a permanently active KRAS protein and, thus, incessantly active signaling pathways [137]. Primary driver mutations of *KRAS* are very rare in GISTs (less than 0.5% of cases). Secondary mutations in *KRAS* occur as an after-effect of imatinib treatment in *KIT/PDGFRA* mutant GISTs. The incidence is unknown [42,138,139,140,141].

Gastrointestinal stromal tumors with a *KRAS* mutation do not respond to imatinib treatment. Sotorasib is a specific KRAS G12C inhibitor, the efficacy of which has been determined in a phase 1/2 clinical trial; however, it included no patients with metastatic, *KRAS* G12C-mutated GISTs [142].

#### 3.2.5. GISTs without KIT/PDGFRA Mutations and with NTRK Alterations

The *NTRK1* proto-oncogene is located on chromosome 1 (q23.1), the *NTRK2* proto-oncogene on chromosome 9 (q21.33), and the *NTRK3* proto-oncogene on chromosome 15 (q25.3) [143,144]. The proto-oncogenes *NTRK1*, *NTRK2*, and *NTRK3* encode a family of receptor tropomyosin kinases—TRKA, TRKB, and TRKC—involved in neuronal development. Rearrangements of the *NTRK1*, *NTRK2*, and *NTRK3* genes with different partner genes result in the formation of a constitutively active (ligand-independent) TK, which thereby leads to the development of several solid cancers. Rearrangements are rare in common cancers and common in very rare cancers [144]. *NTRK* rearrangements are agnostic driver alterations, and NTRK inhibitors are effective in tumors with *NTRK* fusions irrespective of the site of origin of metastatic disease [145,146].

##### Targeted Therapy of GISTs without KIT/PDGFRA Mutations and with NTRK Alterations

The *ETV6-NTRK3* and *LMNA-NTRK1* rearrangements in GISTs have already been described [147,148,149,150]. Gastrointestinal stromal tumors with *NTRK* rearrangements do not respond to treatment with imatinib or sunitinib but do respond to treatment with NTRK inhibitors (larotrectinib and entrectinib). A pooled analysis of three phase 1 and 2 clinical trials on the efficacy and safety of larotrectinib enrolled four patients with metastatic GISTs with *NTRK* rearrangements. All four patients treated with larotrectinib had an objective response [151]. Furthermore, three patients with metastatic GISTs and *ETV6-NTRK3* rearrangement treated with larotrectinib have been reported, all of whom responded to treatment, one with a complete response [152]. In another integrated analysis of three phase 1 and 2 clinical trials on the efficacy and safety of entrectinib, one patient with a metastatic GIST was enrolled, but individual efficacy was not reported [150].

#### 3.2.6. GISTs without KIT/PDGFRA Mutations and with FGFR Alterations

Fibroblast growth factor receptor 1 (*FGFR1)* proto-oncogene is located on chromosome 8 (p11.23), *FGFR2* on chromosome 10 (q26.13), *FGFR3* on chromosome 4 (p16.3), and *FGFR4* on chromosome 5 (q35.2). They encode the FGFR family of proteins, which are transmembrane receptors with an IC tyrosine kinase domain [153]. Their activation depends on the conformation (homo- or hetero-dimerization) and consequent (auto- or trans-) phosphorylation of the kinase domain. Activated FGFR is involved in the RAS/MEK/MAPK and PI3K/AKT signaling pathways, among others [153,154,155]. Constitutive activation and overexpression of FGFR may be due to rearrangements or mutations in *FGFR1–4* [47,154]. Primary *FGFR* driver alterations are very rare events in GIST evolution but have been described as a mechanism of resistance to imatinib in addition to secondary *KIT/PDGFRA* mutations [47,98].

##### Targeted Therapy for GISTs without KIT/PDGFRA Mutations and with FGFR Alterations

Multitargeted TKIs that have been found to be active in cases of *FGFR* driver alterations are the standard systemic treatment for metastatic GISTs (regorafenib), while other multitargeted TKIs are currently in clinical trials in this setting: dovitinib, masitinib, ponatinib, lenvatinib, pazopanib, and nintedanib [47,92]. Selective FGFR inhibitors (erdafitinib, infigratinib, pemigatinib, and futibatinib) are available but have so far not been investigated in relation to GISTs [156].

#### 3.2.7. GISTs without KIT/PDGFRA Mutations and with Very Rare Mutations of Unknown Clinical Significance

Since the introduction of NGS into routine clinical practice, several other rare alterations in various genes have been described in relation to GISTs (*PIK3CA*, *MAX*, *MEN1*, *ARID1A*, *ARID1B*, *ATR CBL*, *LTK*, *MEN1*, *PARK2*, *SUFU*, and *ZNF217*) [28,51,99,157]. Most of them are accompanying (passenger) alterations that may affect the clinical course of the disease. However, as the number of cases described so far is limited, there are no data on the therapeutic implications of these changes.

**Figure 2:**
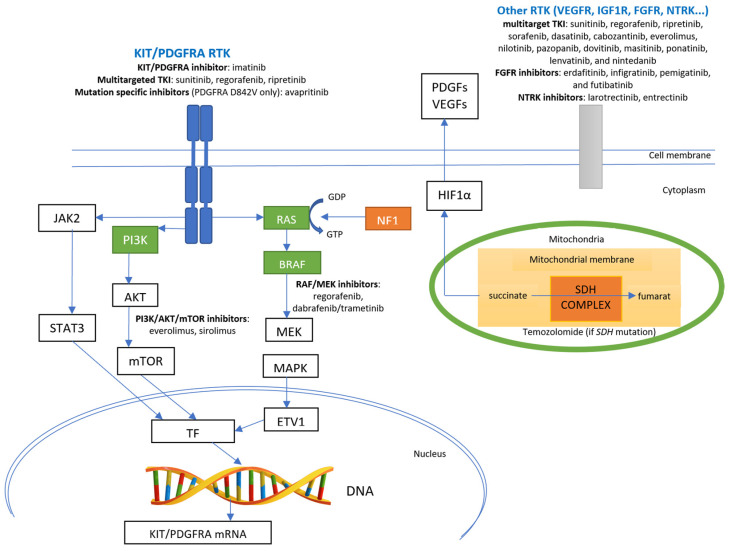
Principal molecular pathways for GISTs and sites of action of specific pathways’ inhibitors. Green boxes indicate kinases with gain-of-function mutations (*RAS*, *BRAF*, and *PI3K* genes); orange boxes indicate proteins with loss-of-function (mutations in the *NF1* gene and loss of function of the SDH complex—either loss of function mutations in genes coding for SDH subunits or epigenetic silencing of the *SDHC* gene). TF—transcription factor; ETV1—ETS variant transcription factor 1; RTK—receptor tyrosine kinase; TKI—tyrosine kinase inhibitor; DNA—deoxyribonucleic acid.

Figure 2 presents the principal molecular pathways for GISTs with the corresponding sites of action of different targeted drugs.

### 3.3. Future Directions

Currently, there are several interventional prospective clinical trials active for GISTs, either in (neo)adjuvant or metastatic settings [158]. Novel drugs or “old” drugs in combination with novel ones are being tested and the results are eagerly awaited. However, a comprehensive review of the novel targeted therapies in development is beyond the scope of this article.

On the other hand, growing research interest has been focused on so-called liquid biopsies. In patients with advanced malignancy, circulating tumor DNA (ctDNA) assays could be utilized to identify clinically relevant mutations for directing targeted therapy. However, the limitations of these assays should be considered carefully [159]. In metastatic GISTs, ctDNA has shown promising applicative value in detecting *KIT* primary and secondary mutations, particularly after progression with imatinib, and in assessing tumor dynamics with serial monitoring [160,161]. Additionally, NGS-based sequencing of ctDNA has demonstrated the potential to foretell the clinical benefit of sunitinib or ripretinib as second-line therapies in patients with advanced *KIT*-mutated GISTs [162].

## 4. Conclusions

Gastrointestinal stromal tumors are rare diseases that vary in their clinical and molecular characteristics. Decision regarding their systemic treatment should be governed by molecular predictive markers (driver alterations). Due to the rarity of the disease and the complexity of treatment necessitated by the variety of molecular characteristics, these patients should be managed in centers that offer the possibility of genuinely comprehensive treatment. The introduction of wide-ranging genomic profiling has improved knowledge about the molecular features of GISTs and opened new perspectives for systemic treatment. Last but not least, increased enrollment of patients in clinical trials is essential, as this leads to improved survival.

## Figures and Tables

**Table 2:** Pivotal clinical trials of TKIs with European Medicines Agency (EMA) approval for the treatment of unresectable or metastatic GISTs.

First Author, Publication Information	Number of Patients, Patient Population	Clinical Phase	Intervention	Molecular Analysis Performed	Primary Endpoint	Response Evaluation Criteria	Results
Demetri et al., NEJM 2002 [76]	147 Advanced GISTs	2	Imatinib 400 mg/d vs. imatinib 600 mg/d	No	ORR	Southwest Oncology Group criteria [105]	ORR: 49.3% (400 mg) vs. 58.1% (600 mg) Estimated 1 y OS: 88%.
Verweij et al., Lancet 2004 [24]	946 Metastatic or unresectable GISTs	3	Imatinib 400 mg/d vs. imatinib 800 mg/d	No	PFS	RECIST 1.0	PFS longer in the group with 800 mg vs. 400 mg, HR 0.82, *p* = 0.026 1 y OS: 85% (400 mg) vs. 86% (800 mg) ORR: 50.1% (400 mg) vs. 54.3% (800 mg)
Blanke et al., JCO 2008 [75]	746 Metastatic or unresectable GISTs	3	Imatinib 400 mg/d vs. imatinib 800 mg/d	No	PFS and OS	RECIST 1.0	Median PFS: 18 m (400 mg) vs. 20 m (800 mg), *p* = 0.13 Median OS: 55 m (400 mg) vs. 51 m (800 mg), *p* = 0.83 No difference in RR between the two arms
Demetri et al., Lancet 2006 [86]	312 Advanced GISTs resistant or intolerant to imatinib	3	Sunitinib 50 mg vs. placebo	No	TTF	RECIST 1.0 or WHO (WHO Handbook for Reporting Results of Cancer Treatment)	Median TTP: 6.3 m (sunitinib) vs. 1.5 m (placebo), HR 0.33, *p* < 0.0001 Median PFS: 5.5 m (sunitinib) vs. 1.4 m (placebo), HR 0.33, *p* < 0.0001 Median OS: not reached. HR 0.49, *p* = 0.007 ORR: 7% (sunitinib) and 0% (placebo), *p* = 0.006
George et al., EJC 2009 [106]	60 Patients with unresectable GISTs resistant or intolerant to imatinib	2	Sunitinib 37.5 mg/d	No	DCR	RECIST 1.0	DCR: 53% Median PFS: 7.8 m Median OS: 24.6 m ORR: 13% 1 y survival rate: 70%
Demetri et al., Lancet 2013 [92]	199 Metastatic or unresectable GISTs resistant to imatinib and sunitinib	3	Regorafenib 160 mg/d vs. placebo	Yes *	PFS	Modified RECIST 1.1 [92]	Median PFS 4.8 m (regorafenib) vs. 0.9 m (placebo), HR 0.27, *p* < 0.0001 HR OS: 0.77, *p* = 0.199 DCR 52.6% (regorafenib) vs. 9.1% (placebo)
Blay et al., Lancet Oncol 2020 [89]	129 Advanced GISTs with resistance or intolerance to imatinib, sunitinib, and regorafenib	3	Ripretinib 150 mg/d + BSC vs. placebo + BSC	Yes (112/129)	PFS	Modified RECIST 1.1 [92]	Median PFS: 6.3 m (ripretinib) vs. 1.0 m (placebo), HR: 0.15, *p* < 0.0001 Median OS: 15.1 m (ripretinib) vs. 6.6 m (placebo), HR 0.36 1 y estimated OS: 65.4% (ripretinib) vs. 25.9% (placebo) ORR: 9%
Heinrich et al. Lancet Oncol 2020 [90]	56 Unresectable *PDGFRA* D842V-GISTs, regardless of previous therapy	1	Avapritinib 300/400 mg/d	Yes (56/56)	ORR	Modified RECIST 1.1 [92]	ORR: 91% CBR: 98% Median DOR: 27.6 m Median PFS: 34.0 m

*—number not provided; ORR—objective response rate, OS—overall survival, PFS—progression-free survival, RECIST—response evaluation in solid tumors, TTF—time to treatment failure, WHO—World Health Organization, DCR—disease control rate, CBR—clinical benefit rate, d—day, m—months, y—year, HR—hazard ratio.
